# Topological and geometric signatures of brain network dynamics in Alzheimer's disease

**DOI:** 10.1002/alz.70545

**Published:** 2025-08-08

**Authors:** Luopeiwen Yi, Michael William Lutz, Yutong Wu, Yang Li, Tananun Songdechakraiwut

**Affiliations:** ^1^ Social Science Research Institute (SSRI) Duke University Durham North Carolina USA; ^2^ Departments of Neurology and Pathology Duke University School of Medicine Durham North Carolina USA; ^3^ Department of Computer Science Duke University Durham North Carolina USA

**Keywords:** Alzheimer's disease, brain network dynamics, network topology, neuroimaging biomarkers, permutation testing, persistent graph homology, sex differences in network structure

## Abstract

**INTRODUCTION:**

This study explores magnetic resonance imaging (MRI) as a promising non‐invasive approach to monitor Alzheimer's disease (AD) and related dementias. We investigate whether dynamic functional connectivity (dFC), which captures time‐varying neural interactions, can reveal sex‐specific brain network disruptions in AD that conventional static connectivity analyses may miss.

**METHODS:**

We analyzed dFC in the Open Access Series of Imaging Studies (OASIS‐3) dataset across three diagnostic groups (normal cognition, mild cognitive impairment, dementia), stratified by sex, and regressed out age. We evaluated group differences using multiple distance metrics sensitive to various aspects of network structure, with statistical significance assessed via permutation testing.

**RESULTS:**

Distinct sex‐specific patterns emerged across diagnostic groups, with each metric sensitive to different aspects of network disruption. Peak connectivity states, rather than mean levels, more effectively reflected brain network dynamics.

**DISCUSSION:**

By emphasizing network dynamics, our findings highlight promising signatures for early detection and longitudinal biomarkers. Future work will explore metrics tailored to specific demographic or clinical subpopulations.

**Highlights:**

- Dynamic connectivity reveals sex‐specific brain disruptions in Alzheimer's disease (AD).
- Peak‐based analysis improves sensitivity over mean‐based connectivity measures.
- Topological and geometric metrics capture distinct network disruptions by sex.
- Mild cognitive impairment shows less consistent connectivity changes due to diagnostic instability.
- Findings support dynamic magnetic resonance imaging (MRI) metrics as early AD biomarkers in future studies.

## 1 BACKGROUND

Alzheimer's disease (AD) is the most common form of dementia, accounting for more than two thirds of cases in individuals aged 65 and older.^1^ It is marked by amyloid beta (Aβ) plaques and tau tangles, which disrupt normal neuronal function and ultimately lead to brain atrophy and behavioral deterioration.^2^ Current diagnostics include biomarker‐based tools such as positron emission tomography (PET) and cerebrospinal fluid (CSF) assays to detect AD pathology,^2^ but these face limitations in routine use. PET involves significant costs and radiation exposure, while CSF analysis requires invasive lumbar puncture. In contrast, magnetic resonance imaging (MRI) offers complementary diagnostic information through a widely available, non‐invasive modality.^3^ Advances in structural, functional, and resting‐state MRI have shown sensitivity to AD‐related brain changes, even in early stages.^4^ Its non‐invasive nature and absence of radiation exposure make MRI particularly suitable for longitudinal monitoring and repeated assessments in clinical trials.^4^ These advantages position MRI as an important component in a comprehensive, multimodal approach to AD diagnosis and monitoring.

Chromosomal sex and age are widely recognized as two of the three greatest risk factors for AD, alongside apolipoprotein E (*APOE*) ε4 genotype.^5^ Sex differences represent a critical but often overlooked dimension in AD research that can significantly impact the interpretation of neuroimaging findings. There is substantial evidence that men and women with AD exhibit different cognitive and psychiatric symptoms, with women showing faster cognitive decline after diagnosis of mild cognitive impairment (MCI) or AD dementia.^6^ Importantly, brain atrophy rates and patterns differ along the AD continuum between the sexes, with women experiencing faster brain atrophy in MCI compared to men.^6^ Therefore, in this study, we stratify our analyses by sex. Besides sex, age represents a fundamental variable in AD research that must be carefully controlled to isolate disease‐specific effects from normal aging processes. Beyond its role as a risk factor, age exerts profound influences on both functional and structural brain connectivity independent of disease status.^7^ Therefore, in this study, we implemented age regression to distinguish between connectivity changes attributable to AD pathology versus those associated with normal aging processes.

Emerging research suggests that AD progression involves disruptions in large‐scale brain network organization,^8^, ^9^ offering a promising diagnostic direction. In particular, dynamic functional connectivity (dFC), which models how patterns of brain communication fluctuate over time, represents a significant methodological advancement beyond traditional static FC.^10^ dFC has shown potential for detecting early alterations in network behavior relevant to AD.^11^, ^12^, ^13^ Static FC measures the average correlation between brain regions across the entire scan, assuming that connectivity patterns remain constant during the acquisition period.^14^ In contrast, dFC captures the time‐varying nature of neural interactions, reflecting the brain's capacity to dynamically reconfigure in response to ongoing cognitive demands.^14^ By accounting for these temporal fluctuations, dFC offers a more nuanced and sensitive characterization of the neural processes underlying AD progression than static approaches.

Connectome studies in AD typically apply graph and distance metrics to static FC. However, AD disrupts both network structure and dynamics.^15^, ^16^, ^17^, ^18^ Extending these metrics to dFC may reveal alterations missed by static analyses. Therefore, a more complete view of AD requires analyzing dFC using these graph and distance metrics. In this study, we analyze dFC across AD stages using persistent graph homology and matrix‐based distance measures. In persistent graph homology, Wasserstein distance measures changes in global network structure over time and is robust to noise.^19^ Standard geometric measures, such as Manhattan,^20^ Frobenius,^21^ and Chebyshev,^22^, ^23^ provide more localized assessments of network dynamics. Nuclear norm captures global patterns of connectivity by emphasizing low‐rank structure.^24^ Spectral distance, based on eigenvalue spectra, captures large‐scale reorganization and shifts in brain networks.^25^, ^26^ Our core hypothesis is that certain distance metrics, when applied to connectome‐based representations of brain organization, with age regression implemented to distinguish AD‐related changes from those due to normal aging, reveal significant differences between diagnostic groups (normal cognition [NC], MCI, and dementia) as well as across sexes.

## 2 METHODS

### 2.1 Dataset

The OASIS‐3 (Open Access Series of Imaging Studies)^27^ dataset is a large‐scale, longitudinal collection of neuroimaging and clinical data aimed at advancing research in AD. It includes data from participants collected over 15 years at the Washington University Knight Alzheimer's Disease Research Center. Designed to support investigations into healthy aging and AD progression, OASIS‐3 provides extensive MRI and PET imaging alongside longitudinal cognitive assessments. It includes an extensive collection of resting‐state functional MRI (fMRI) scans suitable for time series analysis. In addition, OASIS‐3 provides rich clinical assessment data derived from the Uniform Data Set (UDS), which enables the classification of cognitive status. In particular, variables such as cognitive status (NORMCOG), dementia diagnosis (DEMENTED), and impairment without MCI (IMPNOMCI) are used to define four clinical categories: NC, dementia, MCI, and impaired but not MCI. The UDS, developed by the National Alzheimer's Coordinating Center and Alzheimer's Disease Research Centers (ADRC) standardizes clinical and neurological research data across the ADRC network. For this study, the OASIS‐3 data were downloaded on December 11, 2024.

For our analysis, we selected subjects for whom both T1‐weighted (T1w) structural MRI and resting‐state fMRI data were available. The T1w images serve as high‐resolution anatomical references that support spatial alignment and registration of the fMRI data. The resting‐state fMRI sequences, in turn, are used to extract brain activity time series. These scans were downloaded in brain imaging data structure (BIDS) format using the official scripts provided by the OASIS team at https://github.com/NrgXnat/oasis‐scripts.

We carefully refined the fMRI data to retain only the most relevant and temporally consistent information for each subject. Specifically, we selected the latest session and most recent run to align with the most up‐to‐date clinical diagnosis, and included scans within the range of 90 to 600 time points to ensure sufficient temporal coverage. This yielded 1159 unique observations. We excluded 36 observations labeled as “impaired but not MCI,” a diagnostically heterogeneous group not meeting established criteria for MCI, resulting in a final dataset of 1123 observations: 845 NC, 236 with dementia, and 42 with MCI.

The final sample included 619 female and 504 male observations. Among females, there were 495 NC, 105 dementia, and 19 MCI cases; among males, 350 NC, 131 dementia, and 23 MCI cases. The average age was 71.81 years for females (range: 45.66–95.63) and 73.19 years for males (range: 42.50–97.47), as shown in Figure S1 in supporting information.

**RESEARCH IN CONTEXT**

1. **Systematic review**: The authors reviewed literature on functional connectivity alterations in Alzheimer's disease (AD). Emerging evidence suggests that AD progression involves disruptions in whole‐brain network organization. Prior research indicates that age and sex influence AD progression.
2. **Interpretation**: This study introduces an advancing approach for analyzing dynamic functional connectivity (dFC) in AD, leveraging persistent graph homology and geometric distance metrics. These methods capture both global and regional network dynamics and are designed to quantify heterogeneous patterns of brain network reorganization. Findings reveal sex‐specific network disruption and demonstrate that focusing on peak connectivity states increases sensitivity to diagnostic group differences.
3. **Future directions**: Future research will investigate how dFC measures can be aligned with clinical and demographic variables to improve early detection and staging of AD. Longitudinal studies will be essential to determine whether these connectivity‐based markers can predict clinical progression across the AD continuum.

### 2.2 Preprocessing

The goal of preprocessing is to reduce noise and imaging artifacts while preserving meaningful neural signals. The initial step in the preprocessing pipeline involves converting raw imaging data into a standardized format suitable for analysis. In particular, the original digital imaging and communications in medicine (DICOM)^28^ files are converted into the neuroimaging informatics technology initiative (NIfTI) format and organized according to the BIDS^29^ specification, which ensures compatibility across neuroimaging pipelines.

After validating the BIDS‐formatted dataset, we applied fMRIPrep^30^ to perform standardized preprocessing of the resting‐state fMRI data. fMRIPrep is a robust and widely adopted pipeline that integrates tools from multiple neuroimaging software packages, including FSL, ANTs, and FreeSurfer, to conduct a series of preprocessing steps. These steps aim to denoise the input data and extract important information, such as time series.

The preprocessing begins with a series of standardized steps conducted by fMRIPrep, such as head motion correction and slice timing correction, which mitigate motion‐related artifacts and adjust for temporal differences in slice acquisition, respectively. The preprocessed fMRI images are then co‐registered to each subject's T1w anatomical image to improve spatial alignment. Subsequently, both functional and anatomical data are normalized to a standard stereotaxic space (i.e., MNI152NLin2009cAsym) to enable inter‐subject comparisons.

After preprocessing, we extracted region‐wise time series using the automated anatomical labeling (AAL) atlas, which parcellates the brain into 116 regions of interest (ROIs). Confound regression was performed during the time series extraction process using the Nilearn^31^ library, incorporating a standard 36‐parameter model that includes motion estimates, white matter, and CSF signals, along with their derivatives and quadratic terms.

### 2.3 Dynamic functional connectivity construction

**FIGURE 1:**
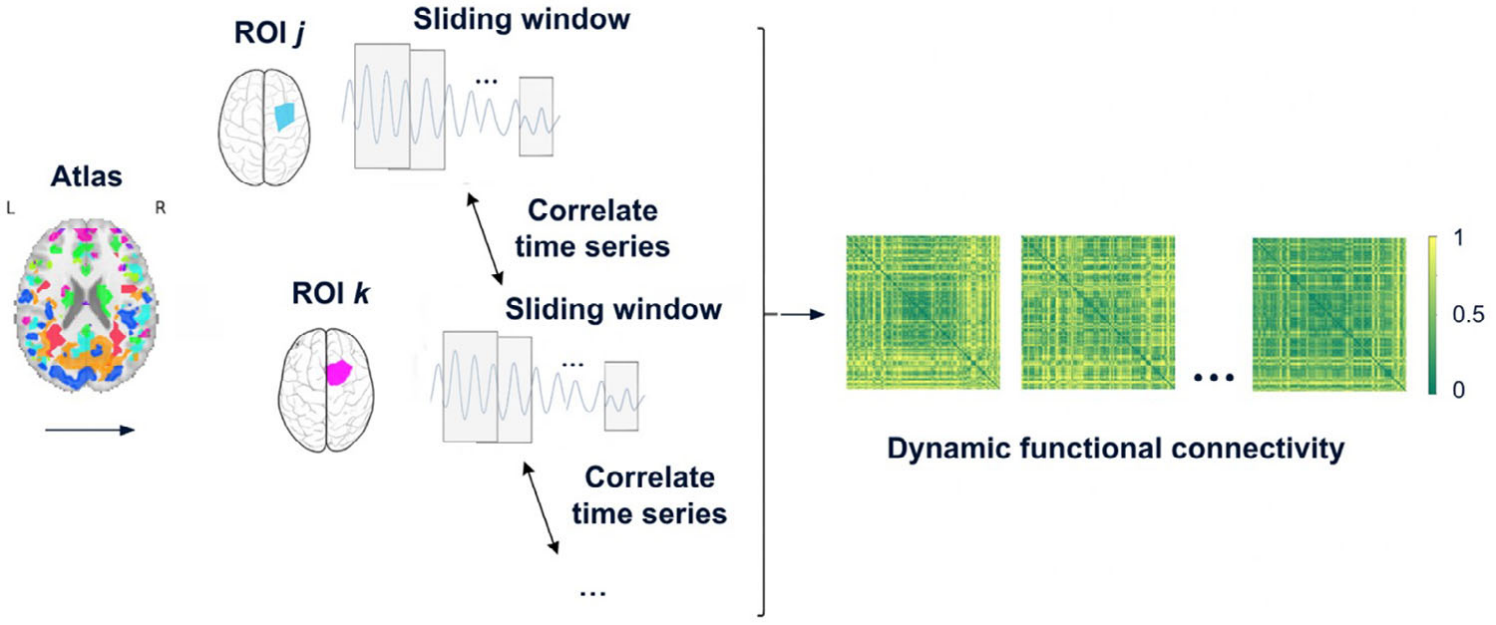
Construction of dynamic functional connectivity across 116 ROIs for a representative subject. ROI, region of interest.

In this section, we present a pipeline for using regional fMRI time series to construct dynamic functional connectivity, as shown in Figure 1. After preprocessing, brain activity is represented as regional time series. To capture temporal connectivity, we used a sliding window approach: for each window $\left(t_{i},t_{i}+s\right)$, we extracted the segment of the time series for each region j and concatenated it into a vector $v_{i}^{\left(j\right)}$. Applying the same window across all M regions yields vectors $v_{i}^{\left(1\right)},\text{…},v_{i}^{\left(M\right)}$. We then quantified connectivity using Pearson correlation. Formally, the Pearson correlation between regions j and k within window i is defined as:

$${\;\rho }_{i}^{\left(jk\right)}=\frac{\sum_{l=1}^{N}\left(v_{il}^{\left(j\right)}-{\bar{v}}_{i}^{\left(j\right)}\right)\left(v_{il}^{\left(k\right)}-{\bar{v}}_{i}^{\left(k\right)}\right)}{\sqrt{\sum_{l=1}^{N}{\left(v_{il}^{\left(j\right)}-{\bar{v}}_{i}^{\left(j\right)}\right)}^{2}}\cdot \sqrt{\sum_{l=1}^{N}{\left(v_{il}^{\left(k\right)}-{\bar{v}}_{i}^{\left(k\right)}\right)}^{2}}}\;$$

where ${\bar{v}}_{i}^{\left(j\right)}=\frac{1}{N}\;\sum_{l=1}^{N}v_{il}^{\left(j\right)}$, ${\bar{v}}_{i}^{\left(k\right)}=\frac{1}{N}\sum_{l=1}^{N}v_{il}^{\left(k\right)}$, and N denotes the number of time points in the window. Brain connectivity was then represented as a graph, with brain regions as nodes and correlation as edges. To capture the strength of connectivity, we applied the absolute value of the correlation. Formally, for window i, we define an M×M connectivity matrix $C_{i}$, whose jk ‐th entry of Ci is given by:

$$\;{\left(C_{i}\right)}_{jk}=\left|\rho_{i}^{\left(jk\right)}\right|\;\;\mathrm{for}\;j\neq k$$

$$\;{\left(C_{i}\right)}_{jk}=0\;\mathrm{for}\;j=k$$

Similarly, by sliding the window forward in time, where $t_{i+1}=t_{i}+c$, we obtained the next window $\left(t_{i+1},t_{i+1}+s\right)$. We then computed the correlation matrix $C_{i+1}$ based on the data within this window. Repeating this process across the entire time series yielded a sequence of connectivity matrices. Specifically, for each subject, we obtained a time‐ordered sequence of connectivity matrices:

$$(C_{1},C_{2},\text{…},C_{T}),$$

where $C_{i}$ denotes the connectivity matrix corresponding to time window i, and T is the total number of windows. This sequence is referred to as dynamic functional connectivity (dFC), as it captures the temporal variation of functional interactions between brain regions.

By adjusting the window size s and the step size c, we controlled the length of each time interval and the overlap between windows. In our analysis, we set the window size to s=5 and the step size to c=2. Because adjacent timestamps in the raw time series are 3 seconds apart, the window size corresponds to a 15 second time interval, and each window is offset by 6 seconds from the start of the preceding window. To ensure consistent dFC estimation across subjects, we clipped each time series to exclude trailing data that did not form a complete window, removed the first and last windows to avoid potential boundary artifacts, and systematically trimmed the remaining data to ensure consistent length across subjects.

### 2.4 Distance metrics for comparing connectivity matrices

To quantify temporal variation in functional brain connectivity, we computed the distance between successive connectivity matrices within each subject's dFC sequence. Let $C_{t\;}$ and $C_{t+1}$ denote two connectivity matrices from adjacent time windows. We measured their dissimilarity using seven distance metrics, each capturing distinct topological or geometric properties of the connectivity patterns, as summarized in Table 1.

**TABLE 1:** Overview of distance metrics: Scope and sensitivity characteristics.

Metric	Local/global	Sensitivity description
Manhattan	Local	Sum of all element‐wise differences
Frobenius	Local	Overall magnitude of all differences
Chebyshev	Local	Maximum single element‐wise difference
Nuclear norm	Global	Sensitive to low‐rank structure, less sensitive to localized changes
Spectral	Global	Compares eigenvalue spectra (whole‐matrix structure)
Wasserstein (0‐homology)	Global	Captures how disconnected brain regions gradually come together to form functional modules
Wasserstein (1‐homology)	Global	Captures how loops of brain connectivity emerge as more connections are included

The resulting time series of connectivity differences for a subject is defined as:

$$\;D_{\mathrm{series}}=\left(D\left(C_{1},C_{2}\right),D\left(C_{2},C_{3}\right),\text{…},D\left(C_{\left(T-1\right)},C_{T}\right)\right)$$

where D(·,·) is a chosen distance function (e.g., Wasserstein distance, spectral distance, etc.). These distances characterize how connectivity changes over time, allowing us to quantify the dynamics of functional brain networks in an interpretable manner across subjects.

#### 2.4.1 Wasserstein distance for 0‐homology and 1‐homology

Persistent graph homology has emerged as a promising tool for understanding, characterizing, and quantifying human connectomes.^32^ It describes interpretable topological invariants, including connected components (0th homology group) and independent cycles (1st homology group or cycle rank) of a graph. Formally, for a connectivity matrix C, we define a binary graph $C_{\varepsilon }$ by thresholding at edge weight ε. That is, $C_{ij}=0$ when $C_{ij}\leq \varepsilon$, and otherwise $C_{ij}=1$. By increasing ε, more edges are removed in a graph filtration:

$$C_{\varepsilon_{0}}⊇C_{\varepsilon_{1}}⊇\text{…}⊇C_{\varepsilon_{k}}$$

with an increasing set of thresholds $\varepsilon_{0}\leq \varepsilon_{1}\leq \text{…}\leq \varepsilon_{k}$ called filtration values. Persistent graph homology tracks how the topological features appear (birth) and disappear (death) across this graph filtration. Each feature is recorded as a point $\left(b_{j},\;d_{j}\right)$ in a persistence diagram, where $b_{j}$ and $d_{j}$ are the birth and death filtration values, respectively.^33^

In this process, as ε increases, the graph becomes sparser, and the number of connected components increases monotonically, while the number of cycles decreases.^34^ Therefore, to summarize the topological changes revealed by persistent graph homology, we only need two sets: the birth filtration values of connected components $B\mathrm{irth}\;\left(C\right)={\left\{b_{j}\right\}}_{j=1}^{M-1}$ and the death filtration values of cycles $D\mathrm{eath}\;\left(C\right)={\left\{d_{j}\right\}}_{j=1}^{1+\frac{M(M-3)}{2}}$, where M is the number of brain regions. Together, these sets form a simplified representation of the persistence diagram, capturing its essential topological features of the graph in a compact and computationally efficient form.

The Wasserstein distance is a prominent measure in persistent homology. It provides a robust measure of dissimilarity between persistence diagrams and is central to the stability theorem.^35^ For two connectivity matrices $C_{t}$ and $C_{t+1}$, the 0‐homology p ‐Wasserstein distance is defined as:

$$D_{p,B}\left(C_{t},C_{t+1}\right)={\left(\sum_{b\varepsilon Birth\left(C_{t}\right)}{\left|b-\tau_{0}^{*}\left(b\right)\right|}^{p}\right)}^{1/p}\;,$$

and the 1‐homology p ‐Wasserstein distance is given by

$$D_{p,D}\;\left(C_{t},C_{t+1}\right)={\left(\sum_{d\varepsilon Death\left(C_{t}\right)}{\left|d-\tau_{1}^{*}\left(d\right)\right|}^{p}\right)}^{1/p}\;,$$

where $\tau_{0}^{*}$ and $\tau_{1}^{*}$ denote the optimal bijections by pairing the l ‐th smallest birth (or death) value in one persistence diagram with the l ‐th smallest value in the other. These distances are well defined because the persistence diagrams being compared are derived from connectivity matrices with the same number of brain regions, ensuring that the corresponding sets of birth and death values have equal cardinality.

#### 2.4.2 Frobenius norm distance

The Frobenius norm distance measures the overall difference between two connectivity matrices by computing the square root of the sum of squared element‐wise differences:

$$D_{\mathrm{Frobenius}}\;\left(C_{t},C_{t+1}\right)=\left|\right|C_{t}-C_{t+1}\;|{|}_{F}=\sqrt{\sum_{i,j}{\left(C_{t,ij}-C_{t+1,ij}\right)}^{2}}.$$

It captures the overall geometric deviation across all region pairs. This distance is intuitive, easy to compute, and well suited for matric comparisons, as studies typically use an identical brain atlas throughout, ensuring consistent matrix dimensionality and node correspondence. However, it may be less sensitive to small, localized changes, as the squaring of small differences further reduces their influence on the overall distance measure. This is particularly relevant in noisy data like fMRI, in which meaningful localized changes can be overshadowed by large, noise‐driven fluctuations that are amplified by the squaring operation.

#### 2.4.3 Manhattan distance

The Manhattan distance measures the total absolute difference between corresponding connections in two connectivity matrices:

$$D_{\mathrm{Manhattan}}\;\left(C_{t},C_{t+1}\right)=\sum_{i,j}\left|C_{t,ij}-C_{t+1,ij}\right|\;.$$

Unlike the Frobenius norm distance, it does not square the differences to amplify large differences. This makes it more responsive to small or localized disruptions in connectivity and less sensitive to outliers that may reflect large noise artifacts. However, it may in turn be more susceptible to small, random fluctuations.

#### 2.4.4 Chebyshev distance

The Chebyshev distance considers only the maximum absolute difference among all elements, making it sensitive to the most significant single deviation:

$$D_{\mathrm{Chebyshev}}\;\left(C_{t},C_{t+1}\right)=\max_{i,j}\left|C_{t,\mathrm{ij}}-C_{t+1,\mathrm{ij}}\right|.$$

This metric focuses exclusively on the largest single deviation between matrix elements, effectively ignoring all smaller differences throughout the network. Such selective sensitivity makes the Chebyshev distance highly responsive to outliers, as it bases its entire calculation on the most extreme change regardless of how small or consistent other variations might be. This property renders the Chebyshev distance particularly useful when extreme variations are meaningful to the analysis, though its value must be weighed against its vulnerability to noise and measurement artifacts.

#### 2.4.5 Nuclear norm distance

The nuclear norm distance is defined as the sum of the singular values of the matrix difference $C_{t}-C_{t+1}$. Formally,

$$D_{\mathrm{Nuclear}}\;\left(C_{t},C_{t+1}\right)=\left|\right|C_{t}-C_{t+1}\;|{|}_{*}=\sum_{i=1}^{r}\sigma_{i}\left(M\right)\;,$$

where $M=C_{t}\;-C_{t+1},$ $\sigma_{i}\left(M\right)$ are the i ‐th singular values of the matrix M, and r is the rank of M.

This metric emphasizes differences in the overall structure or dominant patterns of connectivity rather than individual connections. These dominant patterns are represented by low‐rank structure, in which most of the matrix's information can be captured by a small number of underlying factors or basis vectors. However, it may be less sensitive to localized changes.

#### 2.4.6 Spectral distance

The spectral distance measures the difference in the eigenvalue spectra of two matrices. It focuses on the global structure of the connectivity graph encoded by the matrix. Formally,

$$D_{\mathrm{Spectral}}\;\left(C_{t},C_{t+1}\right)=||\lambda \left(C_{t}\right)-\lambda \left(C_{t+1}\right)|{|}_{2}.$$

Here, $\lambda (C_{t})$ and $\lambda (C_{t+1})$ denote the vectors of eigenvalues of matrices $C_{t}$ and $C_{t+1}$, and $\left||\cdot \right|{|}_{2}$ ​ is the Euclidean norm.

This metric captures global structural differences in the network that might not be apparent from element‐wise comparisons. Rather than being influenced by localized edge changes, spectral distance reflects large‐scale organization as eigenvalues encode overall network energy and stability properties. It is particularly sensitive to alterations in highly connected nodes (hubs) and community structure, making it valuable for detecting fundamental reorganizations in brain networks. This global perspective allows spectral distance to identify meaningful changes in network architecture even when the specific pattern of individual connections may differ.

### 2.5 Sex‐stratified, age‐regressed permutation testing framework

**FIGURE 2:**
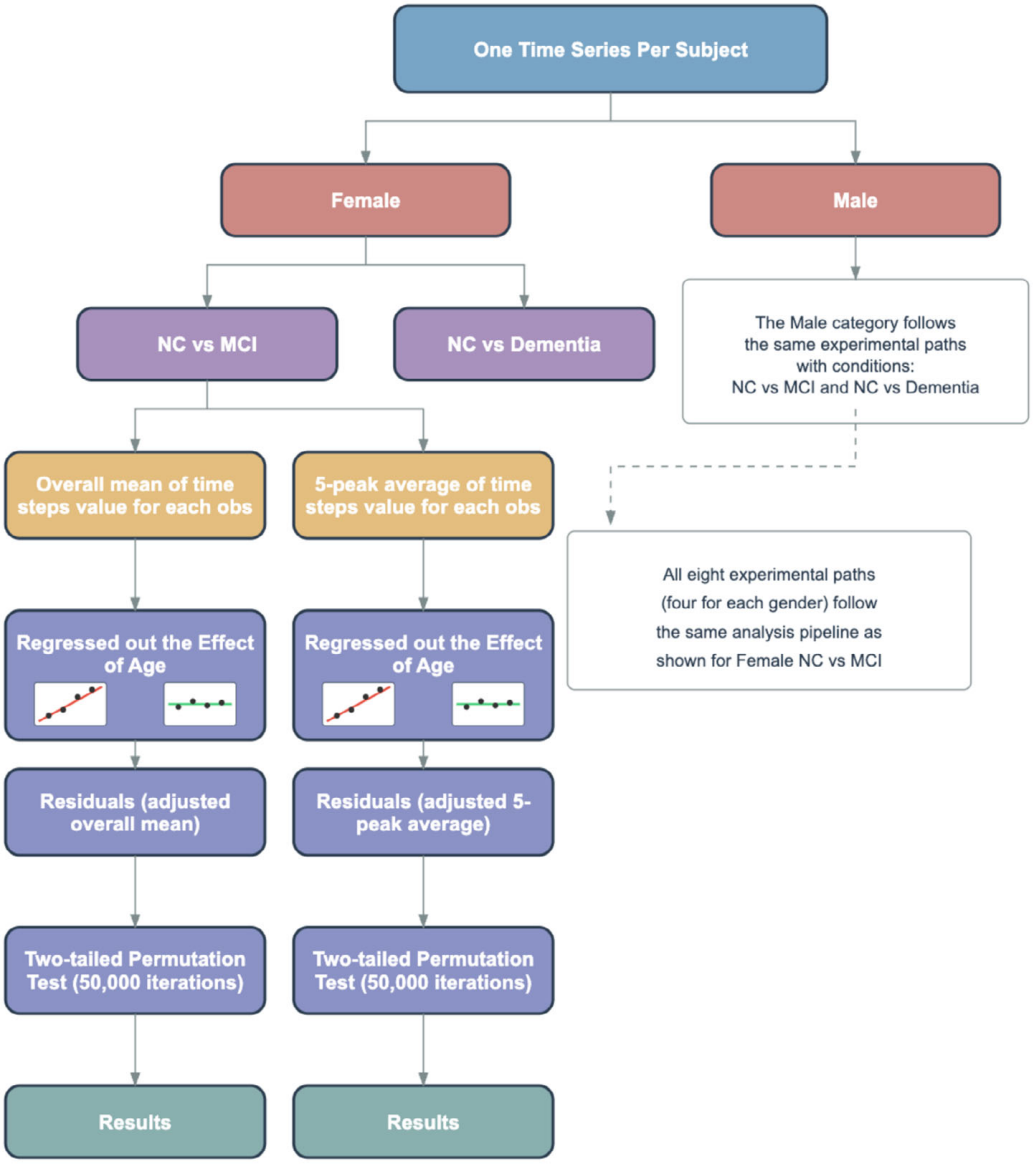
Permutation test experiment pipeline per distance metric. MCI, mild cognitive impairment; NC, normal cognition.

To evaluate whether brain network dynamics differ significantly between cognitive groups (e.g., NC vs. MCI or dementia), we comprehensively conducted permutation tests across sex‐stratified subgroups and pairwise diagnostic comparisons, as shown in Figure 2. Permutation testing is a non‐parametric method that generates a null distribution by repeatedly shuffling group labels. This approach avoids assumptions of normality or equal variance, making it well suited for small sample sizes and complex neuroimaging data. By directly testing group equivalence with minimal assumptions, permutation testing offers robust and valid inference for assessing differences in topological or other geometric distance patterns across cognitive states.^36^, ^37^

In our study, we evaluated seven distance metrics (Frobenius, Manhattan, Spectral, Nuclear, Chebyshev, 0‐homology Wasserstein, and 1‐homology Wasserstein), each representing a different way of quantifying dissimilarity between connectivity matrices. For each distance metric, we treated the analysis as an independent experiment. Within each experiment, we performed four stratified group comparisons, corresponding to two diagnostic contrasts (NC vs. dementia and NC vs. MCI), each evaluated separately for males and females. Given well‐established sex differences in AD, such as faster cognitive decline, greater atrophy in women during MCI, and distinct risk profiles, we stratified analyses by sex to enhance biological interpretability and avoid aggregation bias.^6^, ^38^, ^39^, ^40^, ^41^

To capture both general trends and extreme fluctuations in brain network dynamics, each group comparison included two complementary permutation tests. The first test used the mean across all time points in the dynamic connectivity time series, providing a measure of the average temporal variability in brain network organization and reflecting sustained differences in network dynamics. The second test focused on the average of the five highest peaks in each time series, aiming to detect brief but pronounced disruptions in connectivity. This peak‐based measure is particularly sensitive to short‐lived instabilities or abrupt shifts that may indicate pathological changes associated with AD. Together, these two tests offer a comprehensive assessment of both session‐long fluctuations and transient, extreme changes in brain network dynamics.

For each test, the null hypothesis $\left(H_{0}\right)$ is that there is no significant difference in brain network dynamics, as measured by the distance metric, between the two groups being compared, and that any observed difference is due to random variation. The alternative hypothesis $\left(H_{1}\right)$ is that a non‐random difference exists. Statistical significance was assessed through two‐tailed permutation testing with 50,000 iterations. The high iteration count ensures stable estimation of the *p* value. This approach allows us to assess statistically significant differences in brain network dynamics across cognitive states.

#### 2.5.1 Adjusting for age in group comparisons

Age, a major AD risk factor, interacts with sex and *APOE* ε4^5^ and independently alters brain connectivity,^7^ making it essential to control for. To isolate disease‐specific effects, we applied age regression within each sex group. In the overall mean test, we first computed the mean of all time step values for each individual. To account for the confounding effect of age, we regressed out age‐at‐visit from these individual summary statistics using linear regression, and used the resulting residuals as age‐adjusted measures. The absolute difference in group means (e.g., dementia minus NC) was used as the observed test statistic. For each of the 50,000 permutations, group labels were shuffled while retaining the data structure, and the absolute difference between the permuted group means was calculated. The *p* value was then computed as the proportion of permuted statistics that were greater than or equal to the observed statistic.

The 5‐peak average test followed a similar procedure, except that the summary statistic per individual was the average of their five highest distance peaks across time. This measure captures prominent dynamic fluctuations in brain connectivity that may reflect episodic instability or reorganization. As with the overall mean test, age‐at‐visit was regressed out before comparing the absolute group differences, and the same two‐tailed, 50,000‐iteration permutation framework was applied.

This dual‐testing framework offers a robust, assumption‐free statistical approach tailored to complex time series data. By combining individual‐level summary statistics, age adjustment through linear regression, and rigorous permutation‐based inference, we ensure that the findings are both interpretable and statistically sound, particularly in a context in which traditional parametric assumptions (e.g., normality, homogeneity of variance) may not hold.

#### 2.5.2 Permutation test formula

Recall that $C_{t}$ denotes the connectivity matrix at time window t. The time series of connectivity differences is defined as:

$$\;D_{\mathrm{series}}=\left(D\left(C_{1},C_{2}\right),D\left(C_{2},C_{3}\right),\cdots ,D\left(C_{\left(T-1\right)},C_{T}\right)\right),$$

where D(·,·) is a distance function, applied to quantify the change between consecutive connectivity matrices. Let $X=\{x_{1},x_{2},\text{…},x_{m}\}$ and $Y=\{y_{1},y_{2},\text{…},y_{n}\}$ be the sets of dFC measures derived from $D_{\mathrm{series}}$ for two subject groups (e.g., NC vs. dementia). For the overall mean test, each dFC measure was defined as the mean of all distances between successive connectivity matrices:

$$\;x_{i}=\frac{1}{T-1}\;\sum_{t=1}^{T-1}D\left(C_{t},C_{t+1}\right)$$

capturing the overall temporal variability in connectivity for subject i.

For the 5‐peak test, the dFC measure was instead defined as the average of the five largest values in the distance series:

$$\;x_{i}=\frac{1}{5}\;\sum_{\mathrm{top}\;\mathrm{five}}D\left(C_{t},C_{t+1}\right)$$

reflecting transient but pronounced fluctuations in brain connectivity for subject i. The same procedure was applied to compute $y_{i}\varepsilon Y$.

Let the observed test statistic be

$$\;T_{\mathrm{obs}}=\left|\bar{x}-\bar{y}\right|\;$$

where $\bar{x}$ and $\bar{y}$ denote the sample means of groups X and Y, respectively. We then combine the two groups into a single pooled set:

$$Z=X⋃Y=\left\{x_{1},x_{2},\text{…},x_{m},y_{1},y_{2},\text{…},y_{n}\right\}$$

Randomly permute the labels of Z and compute the test statistic for each permutation $T^{\left(b\right)}$, for b=1,2,…,B, where B is the total number of permutations (e.g., 50,000). That is, we randomly reassign the m and n labels to form new groups $X^{\left(b\right)}$ and $Y^{\left(b\right)}$, and compute the permuted test statistic:

$$\;T^{\left(b\right)}=\left|\bar{x_{\left(b\right)}}-\bar{y_{\left(b\right)}}\right|\;$$

The *p* value is estimated as

$$p=\frac{1}{B}\;\sum_{b=1}^{B}I\left(T^{\left(b\right)}\geq T_{\mathrm{obs}}\right)$$

where

- I(·) is the indicator function that returns 1 if the condition is true and 0 otherwise.
- $T^{\left(b\right)}$ is the permuted test statistic at iteration b.

This is a two‐tailed test, as it evaluates whether the absolute difference between group means is greater than or equal to the values expected under the null distribution.

## 3 RESULTS

We conducted seven independent experiments, each corresponding to a different distance metric. Within each experiment, we performed four stratified group comparisons (based on diagnosis and sex) and applied two complementary permutation test strategies (mean based and peak based), yielding a total of eight hypothesis tests per metric, as shown in Figure 2. To account for multiple hypothesis testing within each distance metric, we applied a Bonferroni correction across the eight subgroup‐level comparisons (four stratified comparisons × two test types), yielding a per‐comparison significance threshold of *α* = 0.006. This correction helps ensure that any detected differences in brain network dynamics across cognitive states are statistically robust and not due to chance.

As shown in Table 2, the NC versus dementia (female) comparison with the 5 peak–based test shows a highly significant difference (*p* value = 0.002), which remains below the Bonferroni‐corrected threshold (α = 0.006). In contrast, the corresponding male comparison shows a weaker effect (*p* value = 0.053), indicating a female‐specific sensitivity in dementia detection. Across comparisons, the 5 peak–based approach consistently yields stronger group differences than the mean‐based method, as indicated by lower *p* values. The Frobenius norm takes a straightforward approach: it compares individual edge weights between groups by summing the squared differences across all connections, without accounting for higher‐order network structure or topology. It does not capture broader network patterns, such as how regions group together or coordinate their activity across the brain. The fact that it still detects a strong female‐specific effect is therefore intriguing. It suggests that the connectivity differences in female dementia may be pronounced even at the level of individual edge weights, pointing to widespread, measurable disruptions in direct pairwise interactions across the brain.

**TABLE 2:** Frobenius dataset analysis results Bonferroni‐corrected significance threshold: α = 0.006.

Comparison	Test type	*p* value
NC versus dementia (male)	Mean based	0.328
NC versus dementia (female)	Mean based	0.063
NC versus MCI (female)	Mean based	0.744
NC versus MCI (male)	Mean based	0.343
NC versus dementia (male)	5 peak based	0.053
NC versus dementia (female)	5 peak based	0.002
NC versus MCI (female)	5 peak based	0.661
NC versus MCI (male)	5 peak based	0.147

Abbreviations: MCI, mild cognitive impairment; NC, normal cognition

As shown in Table 3, the Manhattan distance metric yields results that closely resemble those of the Frobenius norm. In the NC versus dementia (female) comparison using the 5 peak–based test, the result is highly significant (*p* value = 0.002), while the male comparison is weaker (*p* value = 0.045). The fact that a straightforward, edge‐wise metric like Manhattan distance reveals a strong effect is again intriguing. Unlike Frobenius, Manhattan does not amplify large differences or downplay small ones; it treats all deviations proportionally. The fact that it still produces results similar to Frobenius in highlighting a female‐specific pattern suggests that the effect is not being driven solely by extreme changes in a few edges. Instead, it may reflect a robust and widespread shift in connectivity. This raises the possibility that the observed differences are not just noise but represent stable, system‐level changes in network dynamics, possibly pointing to a diffuse yet coordinated reorganization of brain connectivity in female dementia.

**TABLE 3:** Manhattan dataset analysis results.

Comparison	Test type	*p* value
NC versus dementia (male)	Mean based	0.280
NC versus dementia (female)	Mean based	0.067
NC versus MCI (female)	Mean based	0.834
NC versus MCI (male)	Mean based	0.263
NC versus dementia (male)	5 peak based	0.045
NC versus dementia (female)	5 peak based	0.002
NC versus MCI (female)	5 peak based	0.683
NC versus MCI (male)	5 peak based	0.105

Abbreviations: MCI, mild cognitive impairment; NC, normal cognition.

As shown in Table 4, the nuclear norm reveals a highly significant difference between NC and dementia groups in females using the 5 peak–based test (*p* value < 0.001), falling well below the Bonferroni‐corrected threshold (α = 0.006). In contrast, the male comparison shows no significant effect (*p* value = 0.465). This pattern points to a strong, female‐specific disruption in brain network dynamics. What sets the nuclear norm apart is its sensitivity to low‐rank structure in the connectivity matrix. These are patterns in which a small number of dominant modes explain much of the overall connectivity. The nuclear norm specifically highlights changes that simplify or compress the network's structure, where complex patterns are replaced by more generalized patterns of communication. In the context of dementia, this suggests that female patients may experience a collapse or restructuring of network diversity, leading to a more homogenized pattern of brain connectivity. Such a reduction in complexity may reflect a loss of functional specialization, highlighting how dementia reorganizes brain function at a global level in females.[Table alz70545-tbl-0005]

**TABLE 4:** Nuclear norm dataset analysis results.

Comparison	Test type	*p* value
NC versus dementia (male)	Mean based	0.963
NC versus dementia (female)	Mean based	0.110
NC versus MCI (female)	Mean based	0.180
NC versus MCI (male)	Mean based	0.562
NC versus dementia (male)	5 peak based	0.465
NC versus dementia (female)	5 peak based	2.0 × 10⁻⁵
NC versus MCI (female)	5 peak based	0.577
NC versus MCI (male)	5 peak based	0.626

Abbreviations: MCI, mild cognitive impairment; NC, normal cognition.

As shown in Table 5, the 1‐homology Wasserstein metric demonstrates an intriguing male‐specific pattern. The NC versus dementia (male) comparison with the 5 peak–based test shows a significant difference (*p* value = 0.006) that is equal to the Bonferroni correction (α = 0.006). In contrast, the female comparison shows a considerably weaker effect (*p* value = 0.449). The 1‐homology Wasserstein distance quantifies changes in cycles within brain networks, which are common structures used to describe information propagation, robustness, and feedback mechanisms.^42^, ^43^ In this context, cycles represent coordinated, closed‐loop pathways that connect multiple brain regions. A greater number of such cycles typically indicates more strongly connected components and enhanced integrative communication across the brain.^44^ A significant difference in this distance among males with dementia suggests a disruption in the persistence or breakdown of these cycles, pointing to altered higher‐order connectivity. This may reflect a loss of long‐range integrative pathways that support efficient communication and are particularly vulnerable to neurodegenerative damage in the male brain. The fact that these effects on dementia are most pronounced under the 5 peak–based test further implies that such disruptions emerge most clearly during periods of high neural variability, possibly reflecting moments of critical reconfiguration or breakdown in network integrity. These results highlight the potential of persistent graph homology to capture unique, time‐specific, and sex‐dependent alterations in brain network dynamics that may be overlooked by conventional distance metrics.

**TABLE 5 alz70545-tbl-0005:** 1‐homology Wasserstein dataset analysis results.

Comparison	Test type	*p* value
NC versus dementia (male)	Mean based	0.019
NC versus dementia (female)	Mean based	0.685
NC versus MCI (female)	Mean based	0.304
NC versus MCI (male)	Mean based	0.145
NC versus dementia (male)	5 peak based	0.006
NC versus dementia (female)	5 peak based	0.449
NC versus MCI (female)	5 peak based	0.981
NC versus MCI (male)	5 peak based	0.032

Abbreviations: MCI, mild cognitive impairment; NC, normal cognition.

As indicated in Table S1 in supporting information, the 0‐homology Wasserstein metric reveals an intriguing pattern similar to what we observed in 1‐homology Wasserstein metric results. While no comparisons reach statistical significance after Bonferroni correction (α = 0.006), there appears to be a male‐specific sensitivity for dementia detection. The NC versus dementia (male) comparisons show the strongest effects with the 5 peak–based test (*p* value = 0.039), whereas the effect is notably weaker in the female comparison (*p* value = 0.589). This male‐specific pattern may stem from the unique brain topology captured by persistent graph homology when using the 0‐homology Wasserstein distance. Specifically, the Wasserstein distance quantifies differences in the appearance and merging of connected components across a range of graph filtration thresholds. In the context of brain networks, a connected component represents a set of brain regions that are internally connected but disconnected from the rest of the network, offering insight into how localized subnetworks form and integrate as connectivity thresholds vary. The observed sensitivity in males suggests that dementia may disrupt the organization of these components differently in male brains compared to female brains. This may reflect sex‐specific pathways through which neurodegeneration alters whole‐brain network topology.^45^ The stronger effects on dementia observed with the 5 peak–based test suggest that these topological disruptions are temporally localized, emerging during short‐lived, high‐variability periods of brain activity, rather than being distributed uniformly throughout the entire scan.

As indicated in Table S2 in supporting information, the spectral distance metric reveals interesting trends in brain connectivity differences. While no comparisons reach statistical significance after Bonferroni correction (α = 0.006), there appears to be a male‐specific sensitivity for dementia detection. The NC versus dementia (male) comparisons show the strongest effects with the 5 peak–based test (*p* value = 0.009), whereas the effect is notably weaker in the female comparison (*p* value = 0.345). These results align with the fundamental nature of spectral distance as a measure of global network organization. Rather than detecting localized connectivity changes, spectral distance captures large‐scale alterations in network structure through eigenvalue distributions. In dementia, this likely reflects disruptions to major brain hubs and community structures that maintain cognitive function. The stronger signal in males suggests that dementia may cause large‐scale network reorganization patterns in males. The greater sensitivity of the 5 peak–based approach suggests that dementia‐related network disruption in males may occur more abruptly across specific time points. These abrupt changes likely reflect pronounced shifts in the eigenvalue spectra, indicative of large‐scale reorganization of brain network topology.

As indicated in Table S3 in supporting information, the Chebyshev distance metric has no comparison reaching statistical significance. The uniformly high *p* values suggest that this metric may be less sensitive to the types of brain connectivity alterations present in dementia and MCI conditions. This lack of sensitivity likely stems from the fundamental nature of Chebyshev distance, which considers only the maximum difference between corresponding elements in the connectivity matrices while disregarding all other variations. In brain networks affected by dementia or MCI, the pathology appears to manifest as distributed patterns of connectivity changes rather than isolated extreme alterations in specific connections. The uniformly high *p* values across all comparisons suggest that the maximum connectivity differences between groups are not sufficiently distinct to serve as reliable markers of disease. It suggests that neurodegenerative processes affect brain connectivity through subtle, distributed alterations across multiple connections rather than through extreme disruptions to specific pathways that would be preferentially detected by Chebyshev distance.

## 4 DISCUSSION

We identify several key insights from the analysis: metric‐specific sensitivity, the advantages of peak‐based testing, and the instability of MCI diagnosis. These findings underscore the need for neuroimaging methods that are adaptable across populations, sensitive to temporal brain dynamics, and optimized for detecting early or subtle changes in disease progression. Each is discussed below, followed by a discussion of key study limitations.

### 4.1 Differences in sensitivity across distance metrics

The differences we observed in how sensitive each metric is can be explained by how these metrics are designed to measure dFC. Each distance metric captures different aspects of dFC. Some emphasize broad, global patterns across the brain, while others focus more on localized or fine‐grained changes. These underlying differences likely account for the variation in performance we saw across sex and diagnostic comparisons.

The differences in how the metrics performed, with topological distances (i.e., 0‐homology and 1‐homology Wasserstein) and spectral distance detecting more pronounced changes in males, and geometric metrics (Frobenius, Manhattan, nuclear norm) capturing more changes in females, may reflect underlying sex‐specific neurobiological patterns. They also suggest that brain network disruptions appear differently in males and females depending on the scale or pattern being measured. Overall, the geometric metrics supported more widespread but moderate changes in network structure rather than isolated extreme changes in females in contrast to males. This is consistent with a network structure in which females would have several interconnected hubs supporting widespread changes over a larger geographic region in contrast to males with fewer network hubs. Recent work investigating sex‐specific molecular networks and key drivers of AD showed distinct architectures of tau‐based brain‐region connectivity networks in males and females, and further showed that the network architecture in females favored a more rapid spread of neurofibrillary tangles in the brain, consistent with the network structure for females inferred in our study.^46^, ^47^

Recent research demonstrates that centrality, reflecting how hub‐like a region is, plays a critical role in dementia‐related dysfunction, with a stronger association between connectivity changes and regional centrality in females than in males.^48^ Female brains exhibited a greater number of highly central (strongly connected) regions in the healthy state and showed more pronounced dementia‐related disruptions in these hubs compared to males. This suggests that AD in females may preferentially target key hub regions (e.g., within the default mode network), whereas in males, neurodegeneration may involve more distributed or non–hub‐focused alterations. These findings support our results, which indicate that sex‐specific neurodegenerative processes manifest differently in connectivity metrics. While hub‐targeted degeneration in females may result in localized disruptions that are better captured by geometric metrics, males may experience broader topological reorganization, aligning with the increased sensitivity of spectral distance metrics in detecting male‐specific network alterations. Specifically, geometric distance metrics (which are sensitive to large changes in connection strength) captured more changes in females, whereas topological and spectral metrics (which are sensitive to global or structural reorganization) detected more pronounced changes in males.

A promising future direction is to integrate topological and geometric metrics within a unified analytical framework. This combined approach could provide a more comprehensive characterization of brain network changes associated with aging and neurodegenerative diseases. It may also improve diagnostic sensitivity and specificity by enabling the selection of metrics best suited to specific subgroups, such as those defined by sex, age, or clinical stage.

### 4.2 Enhanced sensitivity of peak‐based over mean‐based testing

Our results showed that the 5 peak–based test generally yielded stronger group differences of dementia than the mean‐based test. This suggests that transient, high‐amplitude fluctuations in dFC may be more informative for distinguishing diagnostic groups than time‐averaged connectivity measures. These peak states may reflect brief but meaningful disruptions in brain network coordination that are relevant in early‐stage AD, when neural changes are subtle and variable. These events may serve as potential markers of early neuropathological changes in AD and related disorders. Recent studies support this interpretation. Research has demonstrated that individuals with AD exhibit altered temporal properties of brain network states, such as spending more or less time in specific connectivity configurations.^16^, ^49^ These changes are often missed when data are averaged across the entire scan duration.

A promising future direction is to incorporate peak‐based dFC measures into standard analytic pipelines for functional neuroimaging. Doing so could improve the sensitivity of imaging biomarkers for early detection, monitoring, and stratification of individuals along the continuum from normal cognition to dementia.

### 4.3 Diagnostic instability and heterogeneity in MCI

Across all distance metrics and testing strategies, we observed more robust group‐level differences between NC individuals and those with dementia, compared to those with MCI. Several metrics, including Frobenius norm, Manhattan distance, nuclear norm, and 1‐homology Wasserstein, yielded statistically significant group differences between NC and dementia after Bonferroni correction when using the 5 peak–based test. In contrast, none of the NC versus MCI comparisons reached statistical significance after correction. These results suggest that the network alterations associated with dementia are more substantial and readily detectable by these distance metrics than the potentially subtler changes characteristic of the earlier MCI stage.

This discrepancy likely reflects that brain network alterations in dementia are typically more severe and consistently observed across individuals, compared to the more heterogeneous and subtle changes seen in MCI. A fundamental challenge in studying MCI as a clinical stage is its diagnostic instability. Longitudinal studies have shown that MCI is not always a stable or predictive indicator of progression to dementia.^50^, ^51^ A substantial proportion of individuals initially diagnosed with MCI revert to NC over time.^52^, ^53^ This inherent instability complicates the comparison of MCI groups, often defined at a single time point, with presumably stable NC control groups. By contrast, reversion from dementia to NC is exceedingly rare.^54^ This difference in diagnostic stability helps explain why significant group differences were consistently detected between NC and dementia, but not between NC and MCI.

This instability of the MCI diagnosis introduces heterogeneity that can obscure true group‐level differences in cross‐sectional studies. High reversion rates suggest that MCI groups may include individuals without underlying or progressive neuropathology, thereby diluting group‐level effects. Fluctuations over time imply that a single diagnostic assessment may not accurately reflect an individual's cognitive state or trajectory. This introduces significant noise into group comparisons, reducing power to detect subtle network alterations present in MCI.

Taken together, these findings offer a plausible explanation for the lack of significant NC versus MCI group differences in our analyses. A future direction is to adopt longitudinal study designs, which may provide greater diagnostic precision during the early stages of AD.

### 4.4 Limitations

This study revealed distinct patterns across diagnostic groups and sexes using various distance metrics, but it has a few notable limitations. First, the functional connectivity matrices were constructed using Pearson correlation coefficients between time series of brain region pairs. While Pearson correlation is a well‐established measure in studies of brain functional connectivity due to its simplicity and interpretability, it captures only linear associations and may miss non‐linear interactions. Future work could benefit from exploring alternative connectivity measures, such as mutual information or phase synchronization measures, to detect additional dimensions of network alterations in MCI and dementia. Second, although significant differences were consistently observed between the NC and dementia groups, comparisons involving the MCI group did not remain significant after Bonferroni correction. This may be attributed to the diagnostic heterogeneity and transitional nature of MCI, where individuals may revert to NC or progress to dementia over time. Such variability in clinical outcomes complicates the identification of stable, group‐level network differences, likely contributing to the absence of statistically significant findings in the NC versus MCI comparisons.

### 4.5 Implications and future directions

This study evaluated dFC in the OASIS‐3 dataset to identify sex‐ and diagnosis‐specific differences in brain network dynamics across NC, MCI, and dementia groups. By applying multiple distance metrics and permutation testing, we demonstrate that dFC captures connectivity alterations that reflect sex‐ and diagnosis‐specific brain network dynamics associated with neurodegeneration. The findings further show that the choice of distance metric significantly affects the detection of group‐level differences in dFC across sex and diagnostic categories, and that peak connectivity states more effectively reflect brain network dynamics than mean levels. The variability in metric sensitivity underscores the need to align analytical methods with clinical and demographic factors. These insights may contribute to the development of non‐invasive, imaging‐based biomarkers for early detection and differentiation of AD stages.

## CONFLICT OF INTEREST STATEMENT

The authors declare no competing interests. Author disclosures are available in the supporting information.

## CONSENT STATEMENT

The dataset used in this study is OASIS‐3,^27^ a large longitudinal collection of neuroimaging and clinical assessments. As described in the OASIS dataset documentation: “All subjects participated in accordance with guidelines of the Washington University Human Studies Committee. Approval for public sharing of the anonymized data was also specifically obtained.”^55^ The code used in this study is publicly available at: https://github.com/tinayiluo0322/ad‐dfc‐signatures.

## Supporting information



Supporting Information

Supporting Information
