# Supplementary material for: Topological and geometric signatures of brain network dynamics in Alzheimer's disease
Source: Alzheimers Dement. 2025 Aug 8;21(8):e70545. doi: 10.1002/alz.70545 (PMC12333878; doi:10.1002/alz.70545)
Supplement: Supplementary file 1 — Supporting Information [file ALZ-21-e70545-s001.docx]

Supplementary Figure 1. Distribution of Age by Sex and Label


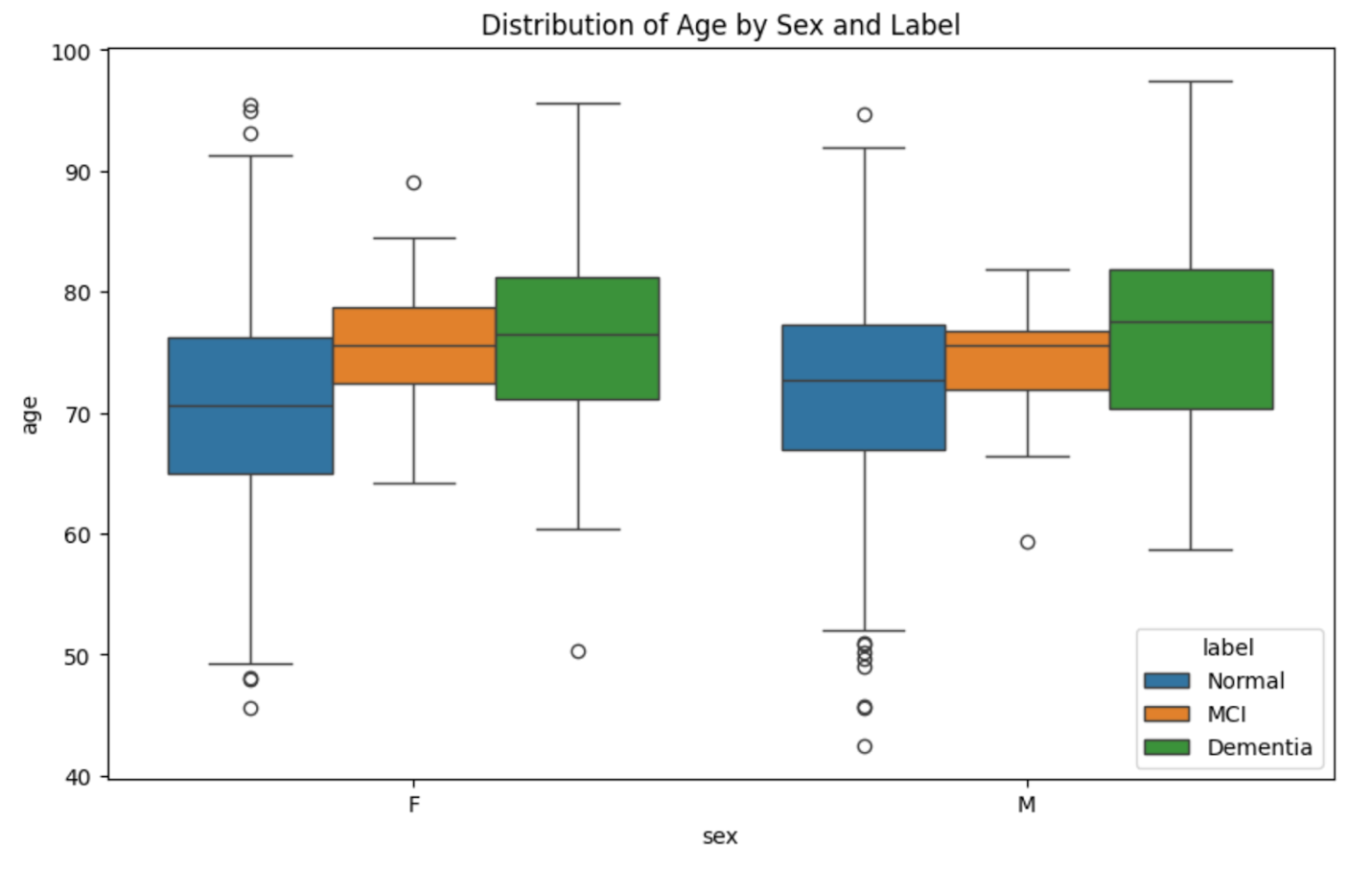


Supplementary Table 1. 0-Homology Wasserstein Dataset Analysis Results

| **Comparison** | **Test Type** | ***P* Value** |
| --- | --- | --- |
| NC vs. Dementia (Male) | Mean-Based | .083 |
| NC vs. Dementia (Female) | Mean-Based | .938 |
| NC vs. MCI (Female) | Mean-Based | .289 |
| NC vs. MCI (Male) | Mean-Based | .199 |
| NC vs. Dementia (Male) | 5-Peak-Based | .039 |
| NC vs. Dementia (Female) | 5-Peak-Based | .589 |
| NC vs. MCI (Female) | 5-Peak-Based | .434 |
| NC vs. MCI (Male) | 5-Peak-Based | .113 |

# Supplementary Table 2. Spectral Dataset Analysis Results

| **Comparison** | **Test Type** | ***P* Value** |
| --- | --- | --- |
| NC vs. Dementia (Male) | Mean-Based | .206 |
| NC vs. Dementia (Female) | Mean-Based | .058 |
| NC vs. MCI (Female) | Mean-Based | .034 |
| NC vs. MCI (Male) | Mean-Based | .403 |
| NC vs. Dementia (Male) | 5-Peak-Based | .009 |
| NC vs. Dementia (Female) | 5-Peak-Based | .345 |
| NC vs. MCI (Female) | 5-Peak-Based | .309 |
| NC vs. MCI (Male) | 5-Peak-Based | .019 |

# Supplementary Table 3. Chebyshev Dataset Analysis Results

| **Comparison** | **Test Type** | ***P* Value** |
| --- | --- | --- |
| NC vs. Dementia (Male) | Mean-Based | .918 |
| NC vs. Dementia (Female) | Mean-Based | .194 |
| NC vs. MCI (Female) | Mean-Based | .125 |
| NC vs. MCI (Male) | Mean-Based | .961 |
| NC vs. Dementia (Male) | 5-Peak-Based | .708 |
| NC vs. Dementia (Female) | 5-Peak-Based | .295 |
| NC vs. MCI (Female) | 5-Peak-Based | .093 |
| NC vs. MCI (Male) | 5-Peak-Based | .918 |
